# The cultural and ecological impacts of aboriginal tourism: a case study on Taiwan’s Tao tribe

**DOI:** 10.1186/2193-1801-3-347

**Published:** 2014-07-08

**Authors:** Tzu-Ming Liu, Dau-Jye Lu

**Affiliations:** Graduate Program of Sustainable Tourism and Recreation Management, National Taichung University of Education, 140 Min-Shen Road, TaiChung, 40306 Taiwan; School of Forestry and Resource Conservation, National Taiwan University, No. 1, Sec. 4, Roosevelt Road, Taipei, 10617 Taiwan

**Keywords:** Tao tribe, Cultural and ecological impact of tourism, Eco-tourism, Evil spirits

## Abstract

We show that tourism activities severely impact the ecology of Orchid Island, its natural resources, and the culture of the Tao tribe. For example, highway widening, in response to the increased traffic volumes caused by tourism, required many Pandanus trees to be cut and removed, which has placed the coconut crabs in danger of extinction. To promote eco-tourism, observation trips to observe Elegant Scops owls and Birdwing butterflies have taken place, which has affected the breeding of these two protected species. The Elegant Scops owls- and Birdwing butterflies-related tourism activities also break the “evil spirits” taboo of the Tao people and have caused the disappearance of the specifications for using traditional natural resources, causing natural ecosystems to face the threat of excessive use. In addition to promoting and advocating aboriginal tourism of the Tao people on Orchid Island, the Taiwanese government should help the Tao people to develop a management model that combines traditional regulations and tourism activities.

## Introduction

The number of global tourists participating in aboriginal tourism has been steadily growing and today is an indispensable category in the tourism industry (Zeppel 2006). With the prevalence of aboriginal tourism, many indigenous people and the areas in which they live are significantly impacted by tourism activities. The Hopi and Acoma Indian reservations in Southwestern United States are a representative example of this concern. The populations of these two areas are approximately 6,000 and 4,000, respectively; however, the regions see 75,000–100,000 and 300,000–400,000 visitors each year (Lew 1999). The ratio of tourists to residents can be greater than several hundred fold, indicating that the demand for aboriginal tourism is significant. The crowds inject money into the local economy. Thus, many aboriginal communities expect to improve their economic environment through the development of tourism, while government agencies also push measures that promote tourism to expand the market of aboriginal tourism and increase the living standards of indigenous people (Butler and Hinch 2007; Whitford and Ruhanen 2010). However, the relationship between tourism growth and the economic development of indigenous people is still controversial (Weaver 2009).

Tourism activities indeed can positively impact aboriginal communities, with these impacts primarily coming from the direct and indirect economic incentives from tourist spending. As an example of a direct benefit resulting from tourism activities, both the number of employed indigenous people and incomes can be increased (Strickland-Munro and Moore 2013). The importance of traditional culture and the Holy Land of the indigenous people may be increased as a result of tourism needs; therefore, these considerations receive more attention and can be more properly preserved (Smith et al. 2009). The ecological environment and conservation of biodiversity in the traditional living areas of indigenous people may receive more adequate protection through the infusion of financial resources for conservation due to tourism activates (Nursey-Bray and Rist 2009; Scherl and Edwards 2007).

Although aboriginal tourism has the aforementioned advantages, some studies also show negative impacts of tourism on indigenous people, including influences on the economy, culture, ecological environment, etc. The business opportunities brought about by tourism can attract the influx of floating populations, which crowd out work opportunities for indigenous people and increase unemployment rates. The increase of tourist spending causes prices to rise at the tourist sites, leading to the inability of the indigenous to be able to afford their daily needs and, consequently, exacerbating the poverty problem. If the employment of indigenous people is limited to the tourism industry and there is a lack of opportunity in other industries, the development of indigenous populations will be more limited (Schellhorn 2010).

Aboriginal tourism also significantly impacts non-economic fields. New modes of economic revenue accompanied by tourism activities may change the traditional social mechanisms of aboriginal tribes. As an example, elders in aboriginal tribes often lack competitiveness in the tourism industry; as a result, their traditional status and power can be undermined (Ishii 2012). Additionally, cultural activities and natural resource management methods that rely on traditional knowledge (such as forbidden areas and related taboos) may therefore decline, and the ecological environment will be affected. Merlan ( 1998) clearly notes that the entry of tourists significantly interferes with indigenous beliefs; thus, their natural resource use methods will be changed. For example, the traditional beliefs held by the indigenous peoples of Edith Falls near Katherine, Australia, include the notion that the Rainbow Serpent resides in some surrounding; they consider those places to be forbidden areas, and tribe members are prohibited from entering to avoid bringing about disaster. However, the local indigenous people believe that the interference of tourists has already led to the disappearance of the Rainbow Serpent; therefore, the activities of the local indigenous people in these forbidden areas has increased (Merlan 1998). This kind of disappearance of the natural resource management culture, resulting from tourism and the increase of human activities in ecologically sensitive lands, will further threaten the ecological environment of the traditional living territory of the indigenous people.

Aboriginal tourism can have both positive and negative impacts on indigenous people and their living environment; therefore, when planning or promoting aboriginal tourism, the impacts of aboriginal tourism always should be investigated, and the results should then be used to adjust the management measures of aboriginal tourism-related activities to take advantage of the benefits and avoid any disadvantages. Although the number of related case studies in each region continues to increase, the complex relationship between tourism development and indigenous development is still difficult to clarify on a per case basis (Weaver 2009). Therefore, it is difficult to directly use other people’s experience to plan and promote aboriginal tourism. One of the prioritized approaches to solve this problem is to investigate the aboriginal tourism impacts in each region.

Investigation of the impacts of Taiwan’s own aboriginal tourism industry on the culture and ecology of the indigenous people in Taiwan is one of the key issues for developing sustainable aboriginal tourism in Taiwan. Taiwan is a multi-ethnic country: there are 14 officially recognized aboriginal tribes, each of which can be further divided into different tribal subgroups. Additionally, the cultures and languages of these subgroups are not the same. Because of the high degree of indigenous cultural diversity, the resources of aboriginal tourism in Taiwan are abundant. In fact, cultural activities and the rituals of indigenous people are already the key items in Taiwanese tourism. The social structure of indigenous people and the ecological resources of their living environment inevitably suffer from the impact of tourism activities. The demands of aboriginal tourism continue to expand, prompting the government to build more public facilities but also causing the exodus of indigenous populations; therefore, it is difficult to continue the cultural heritage, and the habitats of many species will become damaged. Although the negative impacts associated with aboriginal tourism in Taiwan are readily apparent, academic literature presenting in-depth analyses of the causes and results of each negative impact in aboriginal tourism remains limited; additionally, comprehensive studies on tourism, culture, and ecology are even more scarce.

To compensate for the lack of the aforementioned studies, this study uses the Tao tribe on the Orchid Island of Taiwan as the case study to present the impacts of aboriginal tourism on indigenous people and their living environment to promote the understanding of the complex relationship among aboriginal tourism, traditional culture, and ecological protection in academic communities (Buultjens et al. 2010; Strickland-Munro and Moore 2013; Whitford and Ruhanen 2010) and to investigate measures that can reduce the negative impacts of aboriginal tourism in Taiwan. Orchid Island and the Tao tribe were chosen because they have are unique in terms of the natural environment and culture. Additionally, Orchid Island has been open to tourism and in frequent contact with the outside world for less than half of a century; therefore, the impacts of tourism activities on Orchid Island and the Tao tribe can be readily observed and analyzed.

With respect to the ecological environment and cultural activities of Orchid Island and Taiwan, the differences between the Tao tribe and other indigenous tribes in Taiwan are significant. The Elegant Scops owl is endemic to Orchid Island, and the Birdwing butterfly is a protected species in Taiwan and is only present on Orchid Island and Batanes Island. The living culture and rituals, such as underground houses, canoes, and the flying fish festival, are characteristic of the Tao tribe; other aboriginal tribes in Taiwan do not have share these in their culture. However, indigenous communities with similar language and culture to that of the Tao tribe are found on Batanes Island. Because of the unique anthropological nature of the Tao, during the Japanese occupation era, the colonial government identified Orchid Island as a study area and barred people from entering the island. During the early era of the National Government, the military controlled tourist entry to Orchid Island. In 1967, Kaiyuan Harbor was officially opened, and tourism followed. In 1970, when the Orchid Island aerial line was opened, tourism began to increase; the degree of impact on Orchid Island and the Tao tribe caused by the outside world also significantly increased.

With respect to the ecology of Orchid Island and the culture of the Tao tribe, the impacts of tourism on the three protected wild animal species of Taiwan, including Elegant Scops owls, Birdwing butterflies, and coconut crabs, are relatively apparent. These three animals play important roles in the culture of the Tao tribe and are indicators of the traditional biological resources. The strong cultural taboo strictly prohibits members of the Tao tribe from hunting and collecting these; however, these three species have become protected because their population numbers continue to decrease. This change occurred after Orchid Island was opened for tourism; therefore, these three species can be studied to investigate the impacts of tourism activities. Thus, in this study, we select Elegant Scops owls, Birdwing butterflies, and coconut crabs to analyze the impacts of aboriginal tourism on the traditional culture and ecology of indigenous people.

## Introduction to Orchid Island and the Tao tribe

Orchid Island is located off the southeastern coast of Taiwan, with its closest distance to Taiwan Island equal to approximately 80 km. Orchid Island covers an area of approximately 45 km^2^ and is the second largest affiliated island of Taiwan. Its shortest distance to the Batanes Island of the Philippines is only 100 km, and the Kuroshio Current flows from Batanes Island to Orchid Island; therefore, the similarity of species and ecology between Orchid Island and the Philippines is higher than that between Orchid Island and Taiwan. As an example, approximately 29 plant species are common among Orchid Island and Taiwan but are not found in the Philippines, while approximately 32 plants are commonly seen both on Orchid Island and in the Philippines but not on Taiwan. Based on these characteristics of plant composition, Hosokawa ( 1958) suggests that the Neo-Wallace Line should pass between Taiwan and Orchid Island, with Orchid Island instead belonging to the Philippines floristic region rather than the East-Asian floristic region, which Taiwan is in.

In addition to Orchid Island’s similarity to the Philippines in terms of ecological characteristics, the consanguinity and culture of the indigenous Tao tribe on the island are also very similar to that of Filipinos. In fact, the oral history of the Tao tribe mentions that their ancestors were from Batanes Island; the oral literature of Batanes Island also records that the Batanes people migrated to Orchid Island due to tsunami. The dialects currently used by residents in these two places are 60% similar, and the ways in which these peoples use torches to trap and catch flying fish at night are identical. The type of canoes used by the Tao people can be found at Batanes Island. Regardless of historical literature, language, or life style, these two places present many historical links.

The cultural characteristics of the Tao tribe, such as the flying fish festival, canoes, and underground houses, are unique to the Tao tribe in Taiwan; other indigenous tribes in Taiwan do not have such cultural features. The flying fish season represents the core of the life and culture of the Tao tribe; the annual rituals and lifestyles are operated according to the flying fish season. The flying fish season spans from February to August each year, with several important ceremonies taking place during this period, such as mivanwa (flying fish attracting festival) and manoigoyin (end of eating flying fish festival). During the flying fish season, the people are not allowed to catch other than the flying fish and the common dolphinfish; they also have specific regulations for fishing gear and the number of people allowed to fish. During this period, all tribe members must follow traditional norms and avoid violating the related taboos, i.e., must not interfere with the tribe peoples’ lifestyle or affect their personal social status.

Canoes are important tools for earning a living, holding importance as tools allowing the Tao people to go sea fishing and as the symbols of social status. The body of a canoe is assembled from a combination of wood types; based on the size of the body, approximately 21–27 pieces of wood plates are used. Based on position and functional necessity, the wood plates must be made of different materials; for example, Fiji longan is used for the bilge keel, and bread tree is used for the top level of the side panel. No nails are used to fabricate the ship; only wood nogs made of small-leaved Mulberry are used for joining. To avoid leaks, the gaps between plates are stuffed with fibers from the root of *Zanthoxylum integrifoliolum*.

The houses of the Tao tribe consist of three main constructions: a main house, a working room, and a veranda. The primary function of the main house is sleeping, cooking, storage, and rituals. The working room is a vertical wall structure that can avoid rain and wind. The height of its floor is the same as that of the ground outside the house, and it is a place for activities, such as making handicrafts and entertaining guests. The veranda is a small stilt structure, which is 60–90 cm above the ground; here, Tao people chat, work, and rest. The main house is semi-underground; thus, the Tao house is also called an underground house. It reflects the unique culture of the Tao tribe and, as a structural style, was developed to avoid typhoon disturbances. The main house is built approximately 2 m underground with only the roof above the ground, which can effectively defend the house from strong wind damage.

In addition to their practical functions, canoes and houses are symbols of social status among the Tao people. However, their construction must follow strict regulations, otherwise these items will bring disaster to the owners. In addition to those taboos associated with canoes and houses, the Tao tribe also have various life taboos. For example, food cannot be discarded, catching other fish is prohibited during the flying fish season, catching coconut crabs and fish is prohibited when one’s wife is pregnant, and the selection of fish for consumption should be based on social status. These taboos are closely associated with the conservation of ecological resources and the environment.

Using fish as an example, edible fish are divided into groups based on the people who are allowed to eat them. The fish that women are allowed to eat also can be eaten by everybody else; however, some fish are designated for men, and women are not allowed to consume them. Additionally, certain fish are designated for consumption by old men, and neither young men, women, nor children can eat these fish. Generally, the fish for men and the fish for old men are easy to catch; while the fish designated for women are more difficult to catch. Therefore, for a man to feed his whole family, he must spend more effort catching fish for the women, which compresses the time available to catch other fish types. Because the fish designated for men are easily caught, this taboo potentially saves these species from overfishing. That is, the taboo limits the number of people who can eat these fish; therefore, the taboo also acts to prevent the extinction of certain fish species as a result of overfishing.

The ecology of Orchid Island and the culture of the Tao tribe are gradually changing due to frequent contact with the outside world. To improve the lives and living environment of the tribe people, the government has initiated massive construction projects. In turn, these projects have caused forest destruction, water loss and soil erosion, and damage to traditional underground houses. Members of the tribe have moved to the main island of Taiwan because of jobs; the promotion of compulsory education also has reduced the opportunities for children to learn their traditional culture, thus creating barriers to cultural heritage. Tourism activities also have caused negative impacts on ecology and culture.

## Research methods

This study uses triangulation to reduce the bias in the study (Decrop 1999). The triangulation method incorporates different types of methods, data, observers, and theories to verify and determine the sources of data, data collection strategies, and the validity of time and theoretical frameworks. This study uses many methods, such as participatory observation, in-depth interviews, and a literature review, to collect, analyze, and validate the data. The features, purposes of use, and means of implementation of each method are described below.

### Participatory observation

At the start of this study, a participatory observation was performed to allow the researchers first entering the field the opportunity to understand systematically the characteristics of Orchid Island, to explore issues related to the subject of this study. After the complex relationship between tourism and Tao people in Orchid Island has been understood, more specific research questions and feasible research routes can then be further developed. During research, participatory observation also is continuously used to assess the data obtained from the field.

The researchers first performed participatory observation for four days, assuming the identity of tourists, in October of 2011. The purpose of this trip was to understand the actual mode of operation of the tourism industry in Orchid Island; therefore, the researchers did not display their identity, and their itinerary was arranged by eco-tourism management personnel. On this trip, the researchers participated in all of the commercial eco-tourism packages, including snorkeling, climbing Tienchih (Heaven Lake), engaging in night observation, participating in beach activities, tasting ethnic food, and farming in taro fields. These activities allow researchers to observe the styles of both the tourism industry workers on Orchid Island with respect to guiding tourists and the actual travel behavior of tourists.

After conducting their participatory observation using concealed identities, based on the observation data and historical documents, the researchers assessed the main impacts of tourism on the ecology of Orchid Island and developed a follow-up research framework. Based on the identified research topics, in-depth interviews and further participatory observations were then planned. The research methods of the in-depth interviews and literature review are described in the following sections.

In August and October of 2012, this study arranged two field surveys, with the first being one month in duration and the second two weeks in duration. During these periods, several participatory observations were performed to observe how the tourism industry workers guide tourists on Orchid Island and the behavior of tourists. During these two field surveys, the researchers had already presented their researcher identities and the purpose of their research when they entered the field. The eco-cultural tour observed in 2012 was the same as the travel itinerary in 2011; the observed behavior of the tour guide and the behavior of the tourists did not present significant differences.

### In-depth interview method

In the field surveys conducted in August and October of 2012, this study arranged several in-depth interviews. The respondents included the staff from a community association, school teachers, the culture workers of traditional rituals, bed and breakfast owners, and tour guides for eco-tourism. This study used semi-structured interviews: the respondents first expressed their own opinions, and the researchers first listened. During such interviews, researchers must pay attention to whether the research questions have been fully answered. If there is any doubt with respect to the questions during participatory observation, specific questions are then proposed to the respondents to ask for their opinions.

Hence, the goal of this study method was to obtain the cultural meaning of the Tao tribe with respect to the research topic. Through interviews, the process of eco-tourism development on Orchid Island and its impacts on the local culture and ecology were obtained. The indigenous names of the species discussed in this study, the meanings of the names in their ethnic language, the cultural implications of those meanings, and taboos were obtained through in-depth interviews.

### Literature review method

The literature used in this study includes paper documents and documentary films. These data first assisted researchers in understanding the background of the development of Orchid Island and the impact of tourism in the past. Based on the first participatory observation, which was conducted in the field, and the subsequent literature review, the researchers were able to obtain an overview of the development of the relationship between tourism and the Tao people on Orchid Island, allowing the researchers to draft the question items needed for in-depth interviews. Such analytic results can also assist researchers to confirm the selection criteria for their respondents. For example, the school teachers and cultural workers of traditional rituals were selected as interview respondents based on the literature review results. Because the past literature has recorded many of the cultural taboos on Orchid Island, and the Tao people on Orchid Island typically avoid talking about their cultural taboos, only school teachers and the cultural workers of traditional rituals tend to record and explain these taboos due to the necessity of their jobs. Based on the records in historical literature, this study adds these two groups as interview respondents to obtain data related to cultural taboos.

The literature review method also was used to validate the results of the field surveys. Through a comparison between the historical literature and field records, in particular, the ethnic names of species, the cultural meanings of ethnic names, and the taboos associated with species, the consistency of the interview data was confirmed. The biological and ecological data obtained for these species and the data for public works on Orchid Island are primarily based on the records of related government documents and the results of academic works.

### Ethical considerations

The proposal for Taiwan National Science Council (NSC) research funding described the ethical considerations for investigators. The proposal was reviewed and approved by NSC. Participants were well informed about purpose and procedures of conducting interviews, their rights to decline participation, and anonymity of responses.

## Research results

### Elegant Scops owls

#### Ecology

Elegant Scops owls are nocturnal birds and are endemic subspecies of Taiwan. Because they do not exhibit long-distance migration behaviors and only move regionally, their habitat is confined to Orchid Island. The primeval forests and woodlands near the tribal farmland are their main habitats. They select natural or semi-natural tree holes for nest-making, and they eat insects and other invertebrates for food. In addition to snakes, they have no natural enemies.

#### Culture

The Tao people call the Elegant Scops owl “toto’o”, and regard these owls as the incarnation of evil spirits. One reason for this is that the owls are nocturnal and mainly inhabit the deep forest; therefore, the Tao people associate them with evil spirits. The other reason is that Elegant Scops owls will perch on the Indian Barringtonia, and the Tao people regard this tree as a devil tree; therefore, the Tao people think Elegant Scops owls, which make their nests in devil trees, are evil spirits. The Tao people are not allowed to talk about toto’o in their residence, and they will also chase away a toto’o if it tweets near their residence. Because fear of the Elegant Scops owls is deeply rooted in their hearts and the Tao will go to great lengths to avoid them, the Tao people do not hunt Elegant Scops owls, and it is considered a breach of taboo to be near the owl’s habitat.

#### Tourism impacts

The natural enemies of Elegant Scops owls include various kinds of snakes. Because of cultural taboos, the Tao people do not hunt for, encroach on, or interfere with Elegant Scops owls; therefore, the indigenous people on Orchid Island are not a threat to the survival of Elegant Scops owls. However, the number of Elegant Scops owls is low, and they are listed as endangered. After an in-depth analysis of the reason for this, it was determined that the low number of Elegant Scops owls is highly associated with tourism activities. The impacts of tourism activities on Elegant Scops owls are derived from four aspects, including habitat destruction, roadkill, light exposure, and the loss of taboo, each of which is described in the following sections.

One of the main sources of destruction to the Elegant Scops owl habitat is the result of road widening activities. The public facilities on Orchid Island have significantly increased in recent years, and road widening has played a major role in this process. The original roads on Orchid Island were narrow and were unable to bear the tourist crowd in such poor condition. Therefore, many road sections have been significantly widened in recent years. Many of these widening projects are either within or close to Elegant Scops owl habitats, including their original living space and interfering with their activities. With wider roads, vehicle speeds also have increased, resulting in the increase of roadkill problems. Many Elegant Scops owls have died as a result of being run over by automotive traffic; hence, quickly moving cars have become a new threat to the Elegant Scops owl.

Ecological observations of Elegant Scops owls at night are an important aspect of tourism on Orchid Island. Tour guides, narrators, and bed and breakfast owners lead tourists to forests on the island, using searchlights to find the Elegant Scops owls. This tourism activity severely interferes with the routines of the Elegant Scops owls. The exposure to the strong light from the searchlight causes temporary blindness, preventing the birds from being able to fly away. This light exposure is predicted to harm the birds in additional ways, as well. For example, adult birds caring for owlets are likely to be frightened by the tourists and may leave their nests and never return, which would lead to the deaths of owlets.

Elegant Scops owls are regarded as the messengers of evil spirits and thus must not be hunted or interfered with. However, this cultural taboo is gradually loosening due to tourism activities. When promoting owl night visits and expecting eco-tourism to promote ecological conservation, the local tribe people have struggled with their perception of the owls as evil spirits versus sources of income. With assistance from ecological research experts in Taiwan, the Tao have been trained as ecological observation narrators for Elegant Scops owls. This process has made Elegant Scops owl observation the symbol of eco-tourism on Orchid Island. Because Elegant Scops owls have become tourism stars and ambassadors, many Tao people (especially young men and children) no longer regard Elegant Scops owls as devils and have even changed some stories and legends to cater to tourists. Eventually, even the Tao will no longer be able to identify whether these stories are part of their historical culture or not. Whether the transformation of taboos and traditional stories will have further impact on the conservation of Elegant Scops owls and the cultural changes of the Tao people remains for future in-depth observations and studies.

### Birdwing butterflies

#### Ecology

Birdwing butterflies are the largest butterflies of Taiwan; they are only distributed on Orchid Island and are a protected unique species of butterfly on Orchid Island. The larvae only eat *Aristolochia ovatifolia* and *Aristolochia zollingeriana* for food. The distribution of Birdwing butterflies covers the entirety of Orchid Island; their breeding areas and the larvae’s growth areas completely overlap with the distribution areas of *Aristolochia*. The adult worms are usually found at the edge of shrubbery and forest in close proximity to the beach.

#### Culture

Tao people call Birdwing butterflies “pahapahad no anito”, which means the soul or messenger of evil spirits. The larvae of Birdwing butterflies only eat *Aristolochia*; in the past, many *Aristolochia zollingeriana* have been planted in the graveyards of Orchid Island. Therefore, many Birdwing butterflies lay their eggs in the graveyards. The adult Birdwing butterflies preferentially feed on the white flowers of Sea Mango trees; this plant is also usually present in the graveyards of the Tao people. Because the Tao people have observed that Birdwing butterflies commonly fly around the tribe’s forbidden areas, they regard them as souls or messengers of evil spirits. They believe that people who are close to them or catch them will be possessed by evil spirits and will bring misfortunate.

Protected by taboos, Birdwing butterflies originally did not face the danger of extinction. However, affected by the collecting craze of domestic and international butterfly collectors, Birdwing butterflies were captured in large numbers during the 1970s and 1980s. Therefore, at one point in time, these butterflies were endangered. When the population numbers were significantly reduced, the Tao people did not actively enact any Birdwing butterfly conservation programs due to the restriction of cultural taboos; they even rejected conservation assistance from outside institutions. Luckily, after long-term communication and the continuous investment of conservation funding, the current population numbers of the Birdwing butterflies on Orchid Island have increased.

#### Tourism impacts

Although the population of Birdwing butterflies already has increased, they are still endangered, protected by the “Wildlife Conservation Act”. The Birdwing butterfly also is a protected species subjected to limited trade under the International Union for Conservation of Nature (IUCN) and the Convention on International Trade in Endangered Species of Wild Flora and Fauna (CITES). These restrictive conservation regulations indicate that the survival of the Birdwing butterfly population still suffers a high degree of threat. Currently, Taiwanese government strictly limits the collection of Birdwing butterflies, indicating that Birdwing butterflies are subject to other threats, such as tourism activities.

Recent studies have indicated that the main reason for the disappearance of Birdwing butterflies is the disappearance of *Aristolochia zollingeriana*, the main food source for their larvae (Fang et al., 1998, 2001). The wild population of *Aristolochia zollingeriana* is too scarce; currently, the Council of Agriculture has listed the plant as endangered in the wild according to the evaluation standard for species conservation of IUCN. Road construction is one of the primary causes of the rapid decrease in *Aristolochia zollingeriana* habitat. The tourist activities in the habitat of *Aristolochia zollingeriana* include ecological observation, hiking, and passing through, which also affect the growth of *Aristolochia zollingeriana*. Thus, although tourism activities do not pose a direct threat to Birdwing butterflies, they affect the growth of the plant consumed by their larvae, thereby threatening the overall propagation of Birdwing butterflies.

The collapse of the traditional taboo culture also has been impacted by tourist activity and should not be ignored. Because of the prevalence of tourism activities, Birdwing butterflies have become a star species of eco-tourism on Orchid Island. This change has removed the taboo that Birdwing butterflies are messengers of evil spirits. Whether this change is good or bad for Birdwing butterfly conservation remains to be seen. Although Tao people enjoy the benefits of Birdwing butterfly conservation resulting from the tourism revenue, the weakening of the Birdwing butterfly taboo also may weaken the Tao people’s restriction of using Birdwing butterfly habitats. As the tourism activities have become more frequent, the influences of taboos have grown weaker; therefore, the conservation of Birdwing butterfly habitats is even more important today.

### Coconut crabs

#### Ecology

The distribution of coconut crabs is primarily on tropical islands from the Eastern Indian Ocean to the Western Pacific Ocean, including islands such as Fiji, Nauru, Okinawa of Japan, the Philippines, and Taiwan. IUCN listed coconut crabs as endangered species in the Red list categories in 1996. Taiwan also announced in 1996 that they are protected crustaceans according to the “Wildlife Conservation Act”.

The distribution of coconut crabs in Taiwan is primarily in the Pandanus forests of Taitung, Kenting, Green Island, and Orchid Island. The distances associated with coconut crab activities are limited; normally, coconut crabs do not venture more than 100 m. Coconut crabs lay eggs in the sea, where their larvae develop. Young crabs mainly live in coastal areas with high humidity and soft soil. Adult crabs mainly live in rock caves that are more inland, but they also sometimes dig their own holes at the base of the island. Therefore, there are significant differences in habitat distribution for young and adult crabs. Coconut carbs are omnivorous and saprophagous. They eat at night, and their food includes fruits and the rotted leaves of coconut, Pandanus, and peanut trees. On Orchid Island, one of the major food sources of coconut crabs is the Pandanus fruit; however, the Pandanus fruit is one of the most commonly used ethnobotanicals of Tao tribe.

#### Culture

Coconut crab in the Tao language is “tatos” or “mipeyso”, which mean “bare” and “walk backward”, respectively. The former refers to the appearance of the coconut crab, which means a shell-less hermit crab. The social norm (taboo) accompanied by “walk backward” in the Tao language is that pregnant women and their husbands are prohibited from eating coconut crabs; additionally, breast-feeding women are not allowed to eat coconut crabs. If a pregnant woman or her husband violates this taboo and eats a coconut crab, the labor will be difficult, the baby will crawl backward when learning to crawl, and the baby will walk backward, causing mobility impairments. Hence, to understand this taboo, it is important to reference the naming system on Orchid Island because of the deep social and cultural meanings.

Men on Orchid Island must change their names twice in their lifetimes, once at the birth of the first child and for the second time at the birth of the first grandchild. For example, if a man is named A after birth; when the first child of A is born and is named B, then A will be renamed as SymanB, which means “the father of B”. When the first child of B is born and is named C, then SymanB (whose original name was A) will be renamed as SypenC, which means “the grandfather of C”. Therefore, whether an individual is with or without a child affects the social status of a Tao person. If a child has difficulty moving, the reputation of his father and grandfather will also be affected. Therefore, the taboo of “mipeyso” ensures that pregnant women and breast-feeding women, and their family members, do not catch coconut crabs to avoid family shame.

#### Tourism impacts

The level of cultural taboo associated with coconut crabs is relatively low in comparison than those of the Elegant Scops owls and Birdwing butterflies; however, the level of impact of tourism activities on coconut crabs is higher than the impact on either of the other two species. The impact of tourism activities on coconut crabs primarily comes from road construction and roadkill. The construction and widening of the around-the-island highway cut and removed many Pandanus trees, which greatly decreased the food source of the coconut crabs. Road construction also separates the habitats of the coconut crabs, causing habitat fragmentation. Cement guardrails along the road significantly hinder the catadromous migration of the coconut crabs during breeding seasons, which worsens the future survival problems of the coconut crabs.

The widening of roads increases the frequency of coconut crab roadkill events. Coconut crabs move slowly; during the breeding season, female crabs returning to the beach to lay their eggs are frequently run over by cars and motorcycles. With wider roads, the time required for coconut crabs to cross the roads is increased, further increasing the chance that a crossing crab will struck be a vehicle. Additionally, vehicle speeds tend to increase with road width; therefore, cars are less able to avoid coconut crabs in the middle of roads, increasing the chance that they will run over coconut crabs. Therefore, road widening will worsen the roadkill phenomenon, which has become the primary cause of the decreasing coconut crab population.

## Conclusion

This study employed the Tao tribe living on Orchid Island of Taiwan as an example to investigate the impacts of aboriginal tourism on indigenous culture and the natural environment of their living areas. To reduce study bias, this study invoked the triangulation method for qualitative research and used different types of methods to collect, analyze, and validate the data. The methods used included participatory observation, in-depth interviews, and a literature review.

The analytic results showed that tourism activities significantly impact the ecology of Orchid Island and natural resource management practices related to the culture of the Tao tribe. In response to the traffic volume caused by increased tourism, the around-the-island highway was widened. During this process, Pandanus trees were cut and removed, placing coconut crabs in danger of extinction. To promote eco-tourism, trips to observe Elegant Scops owls and Birdwing butterflies are conducted, interfering with the propagation of these two protective species. Elegant Scops owl and Birdwing butterfly tourism activities also break the “evil spirits” taboos of the Tao people, causing gradual disappearance of the regulations of traditional use of natural resources and causing the natural ecology to face the threat of excessive interference.

The decrease of these three species also reflects the decline of the Tao tribe’s traditional culture. The decrease in coconut crabs is associated with the removal of Pandanus trees; the removal of Pandanus trees indicates that the ethnobotanical resources available to the Tao tribe have decreased, while the traditional crafts developed from the Pandanus trees also have disappeared. The decrease in Elegant Scops owls and Birdwing butterflies reflects the weakening of traditional regulations based on taboos. The frequency of Tao people entering ecologically sensitive lands (the forbidden areas) has increased, and the Tao are more willing to lead tourists into these areas, which significantly increases the human disturbance in ecologically sensitive lands.

The results of this study show that aboriginal tourism on Orchid Island has significantly negatively impacted the Tao tribe and the ecology of Orchid Island. Because of tourism, the cultural meanings of the Tao tribe have suffered qualitative changes, and the survival of important species is facing threats due to tourism. In addition to the promotion of aboriginal tourism on Orchid Island, the Taiwanese government should work to actively restore these three protected species; otherwise, the risk of extinction will be significantly increased due to the flourishing tourism industry on Orchid Island. The traditional culture of the Tao tribe also should be actively restored. The Taiwanese government should perform detailed surveys, record the succession of the Tao tribe’s culture, and assist the Tao people in confirming which cultures are traditional norms and which legends are new innovations due to tourism. The Tao people should be assisted in the development of management models that integrate traditional norms and tourism activities to revive their traditional culture and exert the effect of the conservation of biodiversity.
